# The cardioprotective potential of selected species of mistletoe

**DOI:** 10.3389/fphar.2024.1395658

**Published:** 2024-04-11

**Authors:** Beata Olas

**Affiliations:** University of Lodz, Department of General Biochemistry, Faculty of Biology and Environmental Protection, Łódź, Poland

**Keywords:** cardiovascular disease, cardiovascular activity, hypertension, mistletoe, viscotoxin

## Abstract

Mistletoe is an evergreen woody shrub with stems measuring 30–100 cm. It has leathery, yellowish-green strap-shaped leaves, yellowish-green flowers, and bears typical berries. The most common species is *Viscum album* L., mainly present in Europe and Asia. It is commonly known as European mistletoe or simply, mistletoe. Scientific interest in mistletoe was awakened in the XX century. Mistletoe, especially *V. album* L., has historically been used in the treatment and prophylaxis of CVD, with its properties being confirmed in recent studies. This mini-review describes new aspects of the cardioprotective properties of various species of mistletoe, especially *V. album* L. The effect of oral and subcutaneous application of fresh *V. album* L. extracts on blood pressure has been studied in various models; while the data suggests that mistletoe may be a promising herbal extract with cardioprotective properties, the species has only been tested *in vitro* and *in vivo*, on animals. In addition, it is unclear whether the cardioprotective activity of mistletoe may be due to particular chemical components, as the chemical composition of mistletoe extracts can vary depending on *inter alia* the time of harvest, extraction method and plant part. Hence, this activity may instead result from synergistic interactions between various secondary metabolites. Therefore, further studies are needed to identify the mechanisms of action of mistletoe compounds on CVDs, and determine their interactions with other cardioprotective drugs, their metabolic mechanisms, pharamacokinetics and adverse effects. More research is also needed to determine the therapeutic doses of active ingredients for use in clinical trials; this would require an accurate understanding of the chemical composition of extracts from different species of mistletoe (not only *V. album* L.) and from various host trees.

## Introduction

Cardiovascular diseases (CVDs) have been a leading cause of death globally over the past 20 years, according to World Health Organization (WHO). The most common forms of CVD are hypertension, stroke, myocardial infarction and atherosclerosis. CVDs may be induced by various endogenous and exogenous risk factors, including hypercholesterolemia, blood platelet hyperactivation, diabetes, obesity and oxidative stress, as well as lifestyle factors such as smoking and lack of physical activity.

Fortunately, various plant components are known to possess cardioprotective properties, with one such genus being the of mistletoe. For example, a number of studies have found it to demonstrate protective potential against hypertension (Rodriguez-Cruz et al., 2003; Ofem et al., 2007; Radenkovic et al., 2009; Bachhav et al., 2011; 2012; 2015; Sekeroglu et al., 2011; Jung et al., 2013; Karagoz et al., 2016; Ko et al., 2016).

Mistletoe (*Viscum* L.) is a genus of parasitic plants from the *Viscaceae* family, which includes about 113–150 species. The range of the genus covers the tropical and temperate zone of the Old World, with the greatest diversity being in Africa and Asia (45 species), with fewer being observed in Australia (four species). Europe is home to two species, including European mistletoe (*Viscum album* L.), also known as gui, Mistel, vischio and muerdago., but *V. album* L. is the most well-known and studied mistletoe species. In addition to *Viscum album* L., *Viscum coloratum* (Kom.) Nakai (Korean mistletoe), *Viscum shimperi* Engl., *Viscum capense* L. are also well known. These species are semi-parasitic plants which grow on coniferous and deciduous trees and use suckers to take water and mineral salts from the host. They can be most often found on poplars, lindens, birches, oaks, firs and pines (Whiteman, 2023).

Mistletoe has characteristically-branching olive-green stems and thick, leathery, dark-green leaves. In late autumn and winter, spherical, white, viscous-filled berries the size of peas ripen at the ends of mistletoe shoots. These fruits are regarded as a delicacy by waxwings and thrushes. The sticky content of the fruit sticks to the feathers of the bird, allowing the mistletoe seeds to be moved from tree to tree. In folklore, mistletoe is generally known as a talisman of prosperity and good fortune (Nazaruk and Orlikowski, 2016; Szurpnicka et al., 2020; Song et al., 2021).

Mistletoe extracts, especially aqueous extracts, are applied in both traditional and modern medicine. Mistletoe itself has been known and used in traditional folk medicine for hundreds of years; indeed, Luther and Becker (1987) report that *V. album* L. has been studied for more than 2000 years, particularly with regard to its medical properties. It has long been used in the treatment of neurological diseases, including headaches, epilepsy, dizziness and other (Szurpnicka et al., 2019; 2022). Only the leaves and twigs are used for internal purposes, as the fruits contain substances harmful to humans and can only be used externally. However, in folk medicine, mistletoe fruits were used as a remedy for all sorts of skin problems; for example, fruit ointment has been used to treat many ailments including wounds, burns, frostbite, skin tumors, actinic keratosis and dermatoses. Hippocrates used mistletoe to treat diseases of the spleen, while Elder and Pliny used it to treat epilepsy (Szurpnicka et al., 2019).

Since ancient times, the anti-inflammatory, analgesic, antidiabetic, anti-arrhythmic and hypertensive properties of *V. album* L., were widely known in the traditional medicines of Asia, Africa and Europe, and remain in use in various countries. In addition, information about mistletoe is available in the European, French, German and U.S. pharmacopeias (Szurpnicka et al., 2020; Kleszken et al., 2022; Klingemann, 2024). It has been proposed that the therapeutic effect of *V. album* L. could be due to synergistic interactions between the various secondary metabolites present in its leaves (Segneanu et al., 2022). In addition, the mechanisms of mistletoe action act vary and depend on its phytochemical content and distribution (Montoya-Inzunzo et al., 2023).

Two various groups of mistletoe preparations exists: 1) which are applied at a constant dose of lektines (for example, Lektinol^®^, Eurixor^®^, and Cefalektin^®^), 2) which are applied homeopathically or anthroposophical produced mistletoe preparations, including Plenosol^®^, Helixor^®^, Isorel^®^, Iscador^®^, Iscucin^®^, and Abnobaviscum^®^. They are used in cancer treatment (Staupe et al., 2023).

It should also be mentioned that all parts of mistletoe are toxic. They contain viscotoxins and lectins, which are two groups of toxic proteins. For example, the consumption of mistletoe leaves or berries can result in serious stomach problems (Yousefvand et al., 2022).

This mini-review describes new aspects of the cardioprotective properties of various species of mistletoe, especially *V. album* L. The review is based on a corpus of electronic resources including GoogleScholar, PubMed, Scopus, ScienceDirect, and Web of Science (to 15the February, 2024). The following terms were used: “mistletoe,” “*Viscum* L.,” “*Viscum album* L.,” “*V. album* L.,” and “cardiovascular disease.”

## Chemical content of mistletoe

Mistletoe contains various bioactive substances, like alkaloids, terpenes, phenolic compounds, and proteins, associated with its potential biological activities, but its chemical content depends on the time of harvest, the manufacturing process, and species of host tree (Ko et al., 2016). Previous studies have used a range of phytochemical analysis techniques, such as high-performance liquid chromatography (HPLC) with ultraviolet (UV) detection, gas chromatography-mass spectrometry (GC-MS), liquid chromatography-mass spectrometry (LC-MS), and liquid chromatography-tandem mass spectrometry (LC-MS/MS) (Szurpnicka et al., 2019). Phytochemical studies based on high-performance thin layer chromatography (HPTLC) indicate that *V. album* L. contains chemical compounds from different chemical classes (Pfuller, 2000; Nicoletti and Pitera, 2020). The most characteristic group includes viscotoxins (0.05%–0.1%) and lectins, which have apoptotic and cytotoxic properties; however, other compounds were also identified, including monosaccharides, phenolic compounds, especially flavonoids, phytosterols (β-sitosterol, γ-sitosterol, stigmasterol, campesterol, and other), terpenoids, diheptanoids, amines, organic acids, amino acids and mineral salts, such as potassium, zinc and calcium compounds (Thronicke et al., 2022; Montoya-Inzunzo et al., 2023).

With regard to plant part, the leaves of *V. album* L. were found to demonstrate higher total phenolic compound contents (polyphenols and flavonoids: 57.7 mg/g dry extract and 9.5 mg/g dry extract, respectively) compared with the seeds and fruits (5.9–354 μg/g dry extract). These include various flavonoids (rhamnetin, quercetin, isoquercitrin, apigenin, luteolin, rutin, and other) and phenolic acids (chlorogenic acid, isochlorogenic acid, coumaric acid, sinapic acid, cinnamic acid, gallic acid, caffeic acid, vanillic acid, salicylic acid, ellagic acid, and other) (Pietrzak et al., 2014; 2017; Thronicke et al., 2022; Montoya-Inzunzo et al., 2023).

**FIGURE 1:**
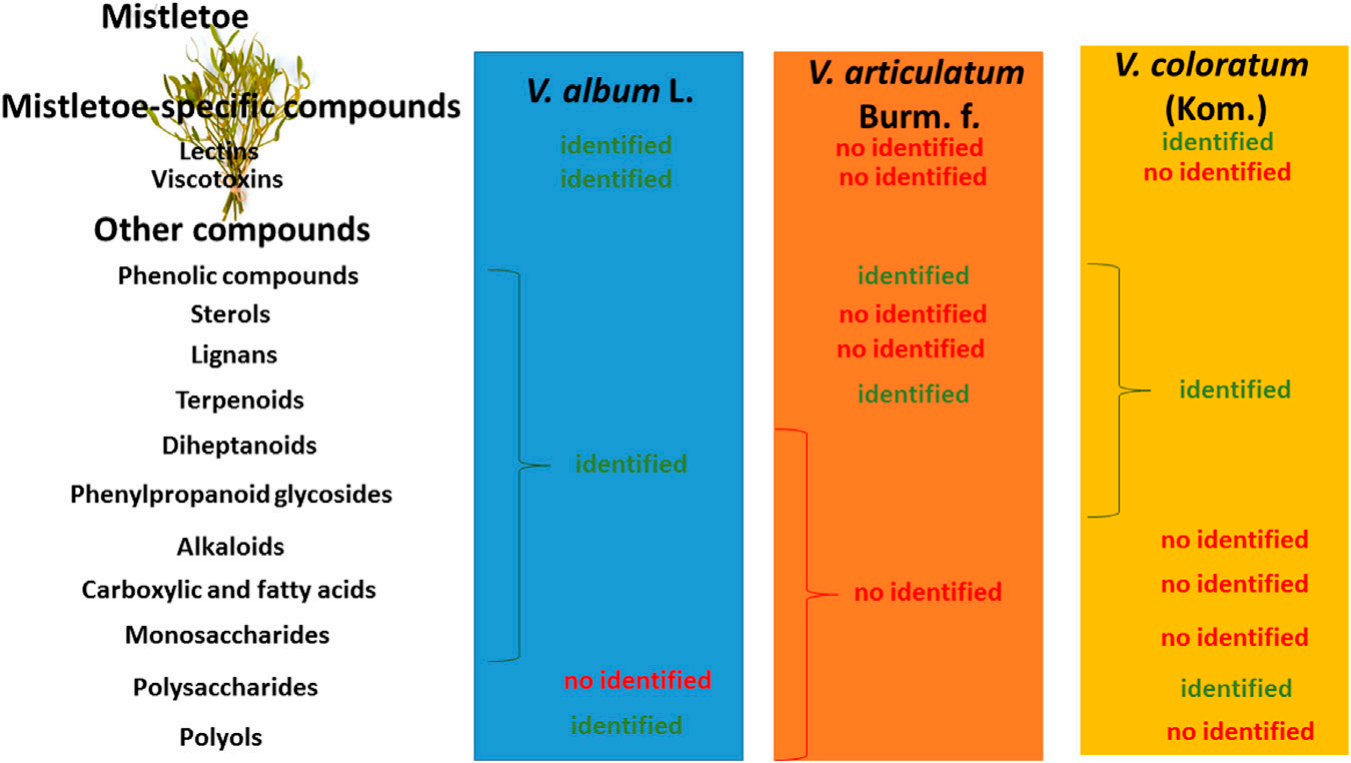
Chemical content of *V. album* L. and other species.

Most importantly, like in some other related species, the fruits and foliage of *V. album* L., contain low-molecular-mass thionin-type proteins, known as viscotoxins, as well as characteristic lectins known as viscolectins. Several viscotoxin isoforms have been described, viz. A1, A2, A3 and A4, with A3 being the predominant isoform (Majeed and Rehman, 2021; Yousefvand et al., 2022; Zhang et al., 2022). Moreover, three types of lectins (termed mistletoelectins I, II, and III) have been identified in *V. album* L. (Yousefvand et al., 2022; Niwa et al., 2003; Ahmad et al., 2018; Szurpnicka et al., 2019; Szurpnicka et al., 2020; de Almeida et al., 2023; Nicoletti, 2023a). The chemical content of *V. album* L. and other mistletoe species are described in Figure 1.

## Cardioprotective action of mistletoe and mechamisms of action of selected chemical components

Various *in vitro* and *in vivo* studies have examined the cardioprotective activity of mistletoe extracts. For example, Suveren et al. (2017) studied the activity of *V. album* L. aqueous and methanolic extracts, prepared from dried leaf, in myocardial ischemia and reperfusion injury in rats. Both tested extracts reduced the extent of infarction compared with untreated controls. The authors propose that these extracts may exert their cardioprotective properties via the nitric oxide/guanylyl cyclase pathway.

Elsewhere, a study based on isolated and perfused Guinea pig hearts found the cardioprotective potential of *V. album* L. aqueous extract to potentially derive from inducible and endothelial nitric oxide. These effects were inhibited by the presence of inhibitors and blockers. Moreover, the tested extract had inotropic effect (Tenorio-Lopez et al., 2006a; Tenorio-Lopez et al., 2006b).

Oral administration of aqueous *V. album* L. leaf extract (250 mg/kg/day) by gavage has also been found to exert a cardioprotective influence on isoproterenol-induced heart failure in rats; which was attributed to regulation of the NO pathway (n = 10) (Karagoz et al., 2016). Other studies indicate that *V. album* L. extract and quercetin supplementation attenuates cyclophosphamide-induced cardiotoxicity, genotoxicity and urotoxicity; this effect has been attributed to anti-inflammatory and antioxidant effects indicated by antioxidant enzyme activity, reduced glutathione level and malondialdehyde production (a parameter of lipid peroxidation) (Sekeroglu et al., 2011). Recently, Nicoletti (2023a) have described the antioxidant properties of *V. album* L. and other species, including various Korean mistletoe, Asian mistletoe, Argentinian mistletoe, Chilean mistletoe, Indian mistletoe, and African mistletoe. In addition, this author suggest that not only phenolic compounds identified in *V. album* L., but also visartisides (a novel class of antioxidant) have antioxidative properties, which may play an important role in the cardioprotective action of mistletoe, although further studies concerning the mechanisms of their action are necessary. Other species of mistletoe, including Chinese mistletoes and Asian mistletoes have also antioxidant potential, which was assigned to phenolic compounds, especially flavonoids (Li et al., 2018; Ahmad et al., 2023). More details about the antioxidant activity of mistletoes are described in the review paper by Nicoletti (2023b).

Various studies have reported anti-hypertensive action of Chilean mistletoes, together with antioxidant activity (inhibition of lipid peroxidation). This activity was investigated in connection with the flavonoid-rich fraction (Dobrecky et al., 2022).

The anti-inflammatory activity of *V. album* L. extracts has been reported in other studies. They have been found to inhibit the action of cyclooxygenase-2 (COX-2), and to destabilize COX-2 mRNA (Elleru et al., 2015; Saha et al., 2015). These anti-inflammatory properties have been associated with antioxidant potential; for example, *V. album* L. contains a number of phenolic compounds, such as flavonoids, which are known to be active scavengers of ROS (Papuc et al., 2010; Vicas et al., 2011; Pietrzak et al., 2014; Saha et al., 2015; Speisky et al., 2022).

Mistletoe may also have anti-obesity potential, which may have an important role in the prophylaxis and treatment of CVDs. *V. album* L. extracts may inhibit adipogenesis and fat accumulation by decreasing the expression of fatty acid synthase, acyl-CoA synthase, acyl-CoA synthetase and other regulators of fatty acid oxidation (Kim et al., 2015).

Panossian et al. (1998) isolated four phenylproponoid glycosides from ethanol extract of *V. album* L. coniferyl alcohol-4-O-β-D-glucopyranoside (coniferin), syringenin 4-O-β-D-glucopyranoside (syringin), coniferylalcohol- and syringe 4-O-β-D-apiofuranosyl (1→2)-β-D-glucopuranosides. All substances were found to inhibit ADP-stimulated blood platelet aggregation *in vitro*. Deliorman et al. (2000) also observed that syringin, coniferin and 5,7-dimethoxy-flavanone-4′-O-β-D-apiofuranyl (1→2)-β-D-glucopuranosides isolated from *V. album* L. induce concentration-dependent contractions in rat aortic rings.

Rodriguez-Cruz et al. (2003) found that the ethanolic extract of the mistletoe *Psittacanthus calculates* (12.5–800 μg/mL), used in Mexican traditional medicine for the treatment of hypertension, has no effect on the basal tone of rat aortic rings (in the absence or presence of indomethacin (an inhibitor of the enzyme cyclooxygenase 1 and 2) or N-nitro-L-arginine methylester (L-NAME), a NO synthase inhibitor); however, low concentrations of this extract (<300 μg/mL) induced a low level of additional tension in both types of rings following precontraction by phenylephrine. In addition, at higher concentrations (>300 μg/mL), the extract relaxed the rings with an intact endothelium. This relaxation was completely reverted by the addition of L-NAME, but not by indomethacin. These results indicate that the endothelium relaxation stimulated by the plant extract was mediated by the synthesis or release of NO.

The antihypertension properties of mistletoe may be also mediated by calcium channel blockade, as indicated in a study of *V. album* L. aqueous leaf extract on rat aortic rings by Mojiminyi et al. (2008) and in a study on rabbit aortic rings by Khan et al. (2016). Ofem et al. (2007) found that *V. album* L. aqueous leaf extract to reduce blood pressure without any alternation in heart rate, and attribute this change to catecholamine-like blocking agents.

Bachhav et al. (2012) also observed that the methanolic extract of *V. articultum* Burm has anti-hypertensive activity in rats when applied at 200 and 400 mg/kg/day, for 4 weeks.

The effect of mistletoe on CVDs may be due to the presence of various phenolic compounds, such as flavonoids, as these have a number of properties that may have cardioprotective effects (Terao, 2023; Thomas et al., 2023). They are known to have anti-platelet, antioxidant and anti-inflammatory properties, and can modulate a range of related signalling pathways, such as the phosphatidylinositol 3-kinase (PI3K)/AKT and mitogen activated protein kinase (MAPK) pathways. For example, Wen-Feng et al. (2006) noted that flavonoids from Chinese *Viscum coloratum* have antiarrhythmic properties in a rat model of arrhythmia induced by aconitine. In addition, flavonoids isolated from *V. coloratum* reduced ischemic myocardial injures in rat model of myocardial infarction by blocking the signaling pathway of platelet-activating factor (Chu et al., 2008).

Some sterols, including β-sitosterol have also demonstrated cardioprotective activity and play an important role in CVDs induced by hypercholesterolemia (Perez-Martinez et al., 2023). The antihypertensive effect of *V. articulatum* Burm. f. has also been attributed to the presence of triterpenoids, such as betulinic acid and oleanolic acid (Bachhav et al., 2012). For example, oleanolic acid isolated from this species significantly decreased systolic blood pressure and cardiac lipid peroxidation in rats (Bachhav et al., 2012). Skrypnik et al. (2022) described the concentration of oleanolic acid in different parts of *V. album* L. (stems: 4.77 ± 1.53 mg/g DW; leaves: 5.02 ± 1.47 mg/g DW, and fruits: 2.62 ± 0.89 mg/g DW).

**FIGURE 2:**
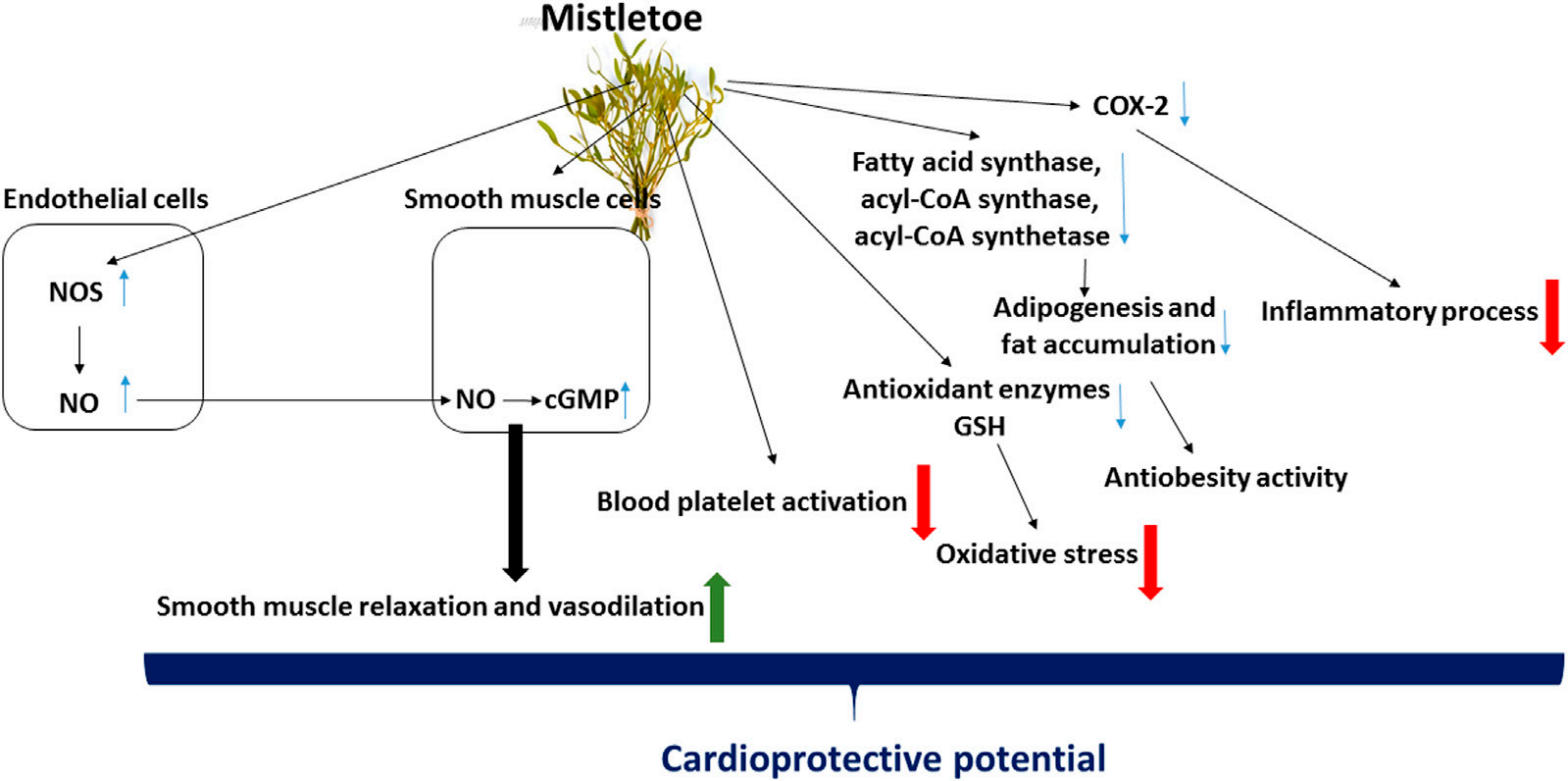
Probable mechanisms of cardioprotective potential of mistletoe. Mistletoe extract may exert their cardioprotective action via the nitric oxide (NO), including inducible and endothelial nitric oxide synthase/guanyl cyclase pathways. The anti-inflammatory properties of mistletoe extracts may be associated with inhibition of cyclooxygenase-2 (COX-2) activity. Mistletoe may also inhibit adipogenesis and fat accumulation. In addition, mistletoe has antioxidant activity and anti-platelet potential. More details are text of manuscript.

In addition, various mistletoe extracts and isolated lectins have demonstrated radical-scavenging properties, and were found to reduce oxidative stress stimulated by reactive oxygen and nitrogen species (Sengul et al., 2009; Kim et al., 2010; 2016; Papuc et al., 2010; Kusi et al., 2015). The probable mechanisms behind the cardioprotective potential of mistletoe are presented in Figure 2.

However, little information exists regarding the cardioprotective activity of isolated chemicals in mistletoe (Panossian et al., 1998; Deliorman et al., 2000). This may suggest that the effects of mistletoe extract result from the synergic activities arising from combinations of different compounds. Mistletoe-specific compounds may also interact with other chemical groups, such as phenolic compounds or phenylproponoid glycosides. In addition, it is possible that the compounds present in mistletoe are also present in other plants, but only mistletoe has the unique combinations that can bestow cardioprotective effects.

## Conclusion

Various historical references indicate the use of mistletoe, especially *V. album* L. in the treatment of CVDs, and these properties have been confirmed in recent years. Recent *in vitro* experiments and animal-based studies on cardioprotective effect of *V. album* L. and other species are presented in Table 1. However, most studies investigating this activity lack the necessary analysis of individual chemical components, and instead relate the activity to *inter alia* total phenolic content and other chemical compounds, including triterpenoids. This is arguably due to the limited approach often taken to the study of herbal and medicinal plants, in which phytochemists and pharmacologists work independently rather than adopting a more holistic approach.

**TABLE 1:** Cardioprotective potential of extracts of *V. album* L. and other species in *in vitro* and animal models.

Part of plant/host tree	Extraction solvent/active constituents	Dose/administration	Biological activity	References
Animal model				
Fresh leaves of *V. album* L./citrus	Aqueous extract/no data	150 mg/kg/day; orally (for 6 weeks)	Antihypertensive properties	Ofem et al. (2007)
Dried leaves of *V. album* L./*Pyrus communis* L. ssp. communis	Aqueous extract/no data	250 mg/kg/day; orally (for 24 days)	Antihypertensive properties	Karagoz et al. (2016)
Dried leaves of *V. album* L./*Quercus* L.	Aqueous extract/no data	500 and 1,000 mg/kg/day	Antioxidant properties	Shahaboddin et al. (2011)
Dried leaves of *V. album* L. and quercetin/*Pyrus communis* L. ssp. communis	Methanolic extract/no data	250 mg/kg/day; orally (for 10 days)	Reducing cyclophosphamide-induced cardiotoxicity	Sekeroglu et al. (2011)
Dried leaves of *V. album* L. and quercetin/*Pyrus communis* L. ssp. communis	Methanolic extract/no data	250 mg/kg/day; orally (for 10 days)	Antioxidant properties	Sekeroglu et al. (2011)
Dried leaves of *V. album* L. and quercetin/*Pyrus communis* L. ssp. communis	Methanolic extract/no data	250 mg/kg/day; orally (for 10 days)	Anti-inflammatory properties	Sekeroglu et al. (2011)
Fresh steams of *V. album* L./no data	Ethanolic, ether and ethyl acetate/no data	3.33 × 10^−5^—1 × 10^−3^ mg/kg; intraperitoneally	Antihypertensive properties	Radenkovic et al. (2009)
Dried herb of *V. articulatum* Burm. F/*Cordia macleodii* (Grift) Hook and Thoms.	Methanolic extract/no data	200 and 400 mg/kg/day; orally (for 4 weeks)	Antihypertensive properties	Bachhav et al. (2012)
Cuticular wax of *V. articulatum* Burm. f/*Cordia macleodii* (Grift) Hook and Thoms.	No data/oleanolic acid	60 mg/kg/day; intraperitoneally (for 15 days)	Antihypertensive properties	Bachhav et al. (2011)
Cuticular wax of *V. articulatum* Burm. f/*Cordia macleodii* (Grift) Hook and Thoms.	No data/oleanolic acid	60 mg/kg/day; intraperitoneally (for 4 weeks)	Antihypertensive properties	Bachhav et al. (2015)
Mistletoe *Psittacanthus calculates* (part of plant—no data)/*Quercus candicans*	Ethanolic extract/no data	12.5–800 μg/mL	Antihypertensive properties	Rodriguez-Cruz et al. (2003)
Dried leaves of *V. coloratum* (Kom.) Nacai/*Quercus candicans*	Aqueous extract/no data	3 g/kg/day; orally (15 weeks)	Anti-obesity properties	Jung et al. (2013)
Dried leaves of *V. coloratum* (Kom.) Nacai/*Quercus candicans*	Aqueous and ethanolic extract/oleanolic acid and betulin	0.2 or 0.6% extract/day; orally (8 weeks)	Anti-obesity properties	Ko et al. (2016)
*In vitro* model				
*V. album* L. (part of plant—no data)/*Quercus* L.	Hydro-alcoholic extract/no data	10–100 μg/mL	Anti-inflammatory properties	Hegde et al. (2011)
*V. album* L. (part of plant—no data)/*Quercus* L.	Hydro-alcoholic extract/no data	25 and 50 μg/mL	Anti-inflammatory properties	Saha et al. (2015)
*V. album* L. (part of plant—no data)/*Armenica vulgaris* Lam.	Methanolic extract/no data	10–100 μg/mL	Antioxidant properties	Yesilada et al. (1998)
*V. album* L. (different parts of plant)/*Molus domestica*	Ethanolic extract/flavonoids	0.05%–0.5%	Antioxidant properties	Holandino et al. (2020)
Leaves and stems of *V. album* L./*Abies alba* Mill	Aqueous extract/flavonoids	-	Antioxidant properties	Pietrzak et al. (2014)
Dried parts of *V. album* L./no data	Ethanolic extract/flavonoids	-	Antioxidant properties	Papuc et al. (2010)

Current studies suggesting that mistletoe may be a promising herbal extract with cardioprotective properties, including antihypertensive activities, have only been based on *in vivo* models (on animals) and *in vitro* models. The compounds responsible for these properties have not been well identified due to the considerable variation present in the chemical composition of mistletoe extracts, which can depend on *inter alia* time of harvest, extraction technique and plant part. The cardioprotective action may result from synergistic interactions of different secondary metabolites rather than individual compounds (Rodriguez-Cruz et al., 2003; Ofem et al., 2007; Rodenkovic et al., 2009; Bachhav et al., 2011; 2012; Karagoz et al., 2016).

Further studies of the effect of mistletoe compounds on CVDs should address their interactions with other drugs with cardioprotective properties, metabolic mechanisms, pharamacokinetics and adverse effects. More research is needed to determine the exact chemical composition of extracts from different species of mistletoe and host trees. These extracts may be used to determine the therapeutic doses of active ingredients for use in clinical trials.

## Abbreviations

COX-2, cyclooxygenase-2; cGMP, guanosine-3′,5′-cyclic monophosphate; CVDs, cardiovascular diseases; GSH, glutathione; L-NAME, N-nitro-L-arginine methylester; MAPK, mitogen activated protein kinase; MDA, malondialdehyde; NO, nitric oxide; PI3K, phosphatidylinositol 3-kinase; ROS, reactive oxygen species; WHO, World Health Organization.
